# Comparison of clinical behavior between mucinous ovarian carcinoma with infiltrative and expansile invasion and high-grade serous ovarian carcinoma: a retrospective analysis

**DOI:** 10.1186/s13000-022-01195-7

**Published:** 2022-01-20

**Authors:** Taira Hada, Morikazu Miyamoto, Hiroki Ishibashi, Hiroko Matsuura, Soichiro Kakimoto, Hideki Iwahashi, Hitoshi Tsuda, Masashi Takano

**Affiliations:** 1 Department of Obstetrics and Gynecology, National Defense Medical College Hospital, 3-2, Namiki, Saitama 359-8513 Tokorozawa, Japan; 2 Department of pathology, National Defense Medical College Hospital, Saitama 359-8513 Tokorozawa, Japan

**Keywords:** Ovarian mucinous carcinoma, Infiltrative invasion, Expansile invasion, Ovarian high-grade serous carcinoma, 2020 World Health Organization

## Abstract

**Background:**

The aim of this study was to evaluate the clinicopathological factors and prognosis of mucinous carcinoma (MC) with infiltrative invasion, MC with expansile invasion, and high-grade serous carcinoma (HGSC).

**Methods:**

Cases of MC and HGSC between 1984 and 2019 were identified. The clinicopathological factors and prognosis of MC with infiltrative invasion or expansile invasion and HGSC were retrospectively compared. Although our present study included cases in our previous studies, we extended observational period when analysis was performed. Accordingly, our study added increased cases and survival analysis was newly conducted.

**Results:**

After pathological review, 27 cases of MC with infiltrative invasion, 25 cases of MC with expansile invasion, and 219 cases of HGSC were included. MC had a better prognosis in terms of progression-free survival (PFS, *p* < 0.01) and overall survival (OS, *p* < 0.01) than HGSC for all International Federation of Gynecology and Obstetrics (FIGO) stages; however, multivariate analysis did not show statistical differences in PFS and OS. There were no statistically significant differences in PFS and OS for all FIGO stages between MC with infiltrative invasion and HGSC. However, in cases with FIGO stages II to IV, MC with infiltrative invasion had worse PFS (*p* < 0.01) and OS (*p* < 0.01) than HGSC. In univariate analysis, MC with infiltrative invasion was a worse prognostic factor for PFS (hazard ratio [HR] 2.83, *p* < 0.01) and OS (HR 3.83, *p* < 0.01) than HGSC. Compared with HGSC, MC with expansile invasion had better PFS (*p* < 0.01) and OS (*p* < 0.01). Multivariate analysis demonstrated that MC with expansile invasion was a better prognostic factor for PFS (HR 0.17, *p* < 0.01) and OS (HR 0.18, *p* = 0.03) than HGSC.

**Conclusions:**

Compared to the prognosis of HGSC, that of MC was different according to the invasive pattern and FIGO stage. Therefore, future study may be needed to consider this association.

## Background

Epithelial ovarian carcinomas (EOCs) are the fourth most common cause of female cancer-related deaths in the developed world [1]. The standard treatment for EOCs is cytoreductive surgery and a combination of platinum- and taxane-based chemotherapy [2]. For EOCs, histological subtypes, residual tumor at cytoreductive surgery, International Federation of Obstetrics and Gynecology (FIGO) stage, and chemosensitivity are important prognostic factors [3–5]. In particular, several histological subtypes exhibit different clinical behaviors. Therefore, histological subtypes are important factors in the management of EOCs.

Among EOCs, the incidence of mucinous carcinoma (MC) ranges from 3 to 11% [6, 7]. The many cases were discovered at earlier FIGO stage and the prognosis of MC at earlier stage was better, but MC with advanced FIGO stage had worse prognosis due to poorer response to conventional chemotherapy [3, 7, 8]. Recently, the invasive patterns of MC have been recognized as an important factor related to tumor aggressiveness, FIGO stage, and prognosis [9–12]. MC with infiltrative invasion is a worse histological subtype than MC with expansile invasion.

High-grade serous carcinoma (HGSC) is the most prevalent histological subtype, with an incidence rate of 70–80% [13]. HGSCs with an advanced FIGO stage are highly sensitive to conventional chemotherapy. Even if tumors respond to conventional chemotherapy, most of them recur [3, 13, 14]. The clinical comparison with HGSC is considered to be important to predict the clinical behavior [15, 16]. Therefore, many studies have demonstrated differences in clinical behavior between MC and HGSC [17–28]. However, there were some discrepancies in the results for each study. We assumed this cause were not to consider the invasive patterns of mucinous carcinoma. In addition, few studies have examined the clinical differences between MC with infiltrative invasion or MC with expansile invasion and HGSC.

Therefore, this study aimed to evaluate the clinicopathology and prognosis of MC with infiltrative invasion, MC with expansile invasion, and HGSC.

## Methods

**Fig. 1:**
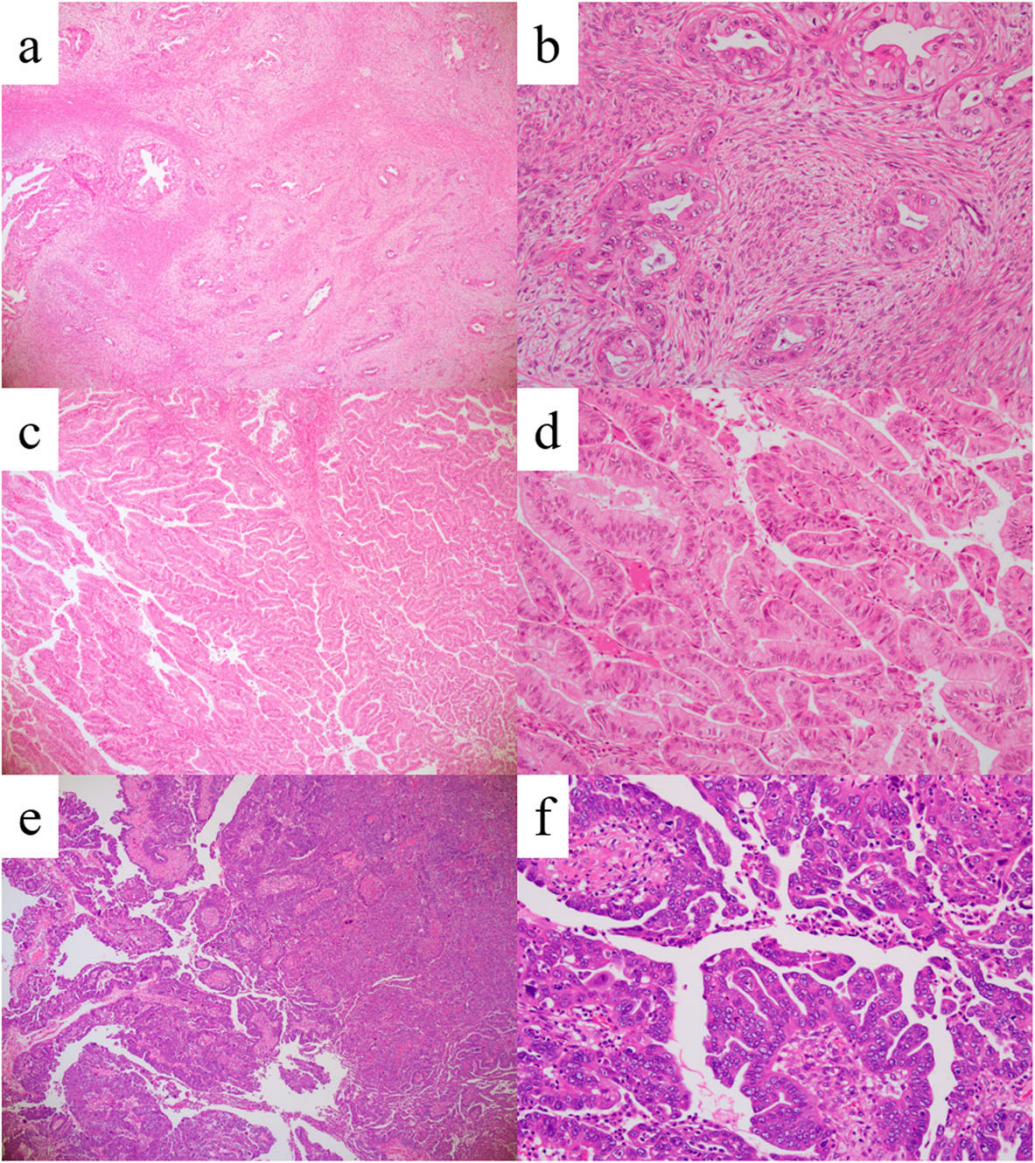
Representative images of mucinous carcinoma (MC) with infiltrative invasion and MC with expansile invasion. **A** MC with infiltrative invasion was characterized by obvious evidence of destructive stromal invasion. (x40) (**B**) Stromal invasion consisted of malignant glands, cell nests, and a desmoplastic stromal reaction (x200). **C** MC with expansile invasion was marked glandular crowding, creating a labyrinthine appearance with little intervening normal ovarian stroma (x40). **D** Back to back malignant glands were seen in MC with expansile invasion (x200). **E** HGSC was characterized by solid, papillary, labyrinthine with slit-like spaces, glandular, or cribriform architecture (x40). **F** Large and markedly atypical nuclei, with high mitotic activity including atypical mitoses, were seen (x200)

Patients with MC and HGSC who underwent primary debulking surgery between 1984 and 2019 at National Defense Medical College were identified. Cases without clinical information and surgical tissue sample, those with a prior history of chemotherapy, and those complicated with other carcinomas were excluded. Pathological review was conducted using the 2020 World Health Organization criteria [29], and cases of MC with infiltrative and expansile invasion and HGSC were included in our analysis. Briefly, the definition of MC with infiltrative invasion was tumors composing of gastrointestinal cells accompanied by destructive stromal invasion of malignant glands with a desmoplastic reaction. Also, the definition of MC with expansile invasion was tumors composing of gastrointestinal cells characterized by a marked glandular crowding with stromal intervening and labyrinthine appearance. Moreover, the definition of HGSC was tumors characterized by cells with slit-like spaces and papillary, glandular and cribriform areas, which were accompanied by numerous and atypical mitoses, or variably conspicuous psammoma bodies. Representative images of MC with infiltrative invasion were shown in Fig. 1 (A) (x40) and 1 (B) (x200), those of MC with expansile invasion were shown in Fig. 1 (C) (x40) and 1 (D) (x200), and those of HGSC were shown in Fig. 1 (E) (x40) and 1 (F) (x200).

Clinical information was collected from medical records. Staging was performed using the 2014 FIGO criteria [30]. Data on residual tumors were obtained from the operation record of the primary surgery. The Response Evaluation Criteria in Solid Tumors, version 1.1, criteria were used to evaluate the efficacy of chemotherapy [31]. Progression-free survival (PFS) was defined as the period from the day of primary surgery to the day of death or disease recurrence or progression. Overall survival (OS) was defined as the period from the day of primary surgery to the day of death or the last follow-up.

Statistical analysis was performed using JMP Pro 14 software (SAS Institute Inc., Cary, NC, USA). Our present study included cases in our previous studies [12, 16]. However, observational period was extended compared with previous reports when analysis in our present study was performed. Accordingly, increased cases were added to our present study and survival analysis was newly performed.

The chi-square test, Fisher’s exact test, and the Mann–Whitney U test were used to evaluate the clinical significance of clinicopathological factors. PFS and OS curves were generated using the Kaplan–Meier method. The survival distribution was compared using the log-rank test. Univariate and multivariate analyses were performed using Cox proportional hazard regression models. Variables with statistical significance in univariate analysis were included in multivariate analysis by backward stepwise selection. The level of statistical significance was set at *p* < 0.05.

## Results

The median follow-up period was 54 months (range, 1–284 months). The mean maximum diameters of ovarian tumor of MC and HGSC were 16.3 centimeters (range, 4-40 centimeters) and 9.0 centimeters (range, 3-30 centimeters), respectively. The median number of sample sections for ovarian tumor of MC and HGSC were 10 (range, 4-22) and 7 (range, 1-24), respectively. Based on the initial pathological diagnosis, we identified 336 cases: 84 cases of MC and 252 cases of HGSC. After pathological review, we included 271 cases: 27 cases of MC with infiltrative invasion, 25 cases of MC with expansile invasion, and 219 cases of HGSC.

**Fig. 2:**
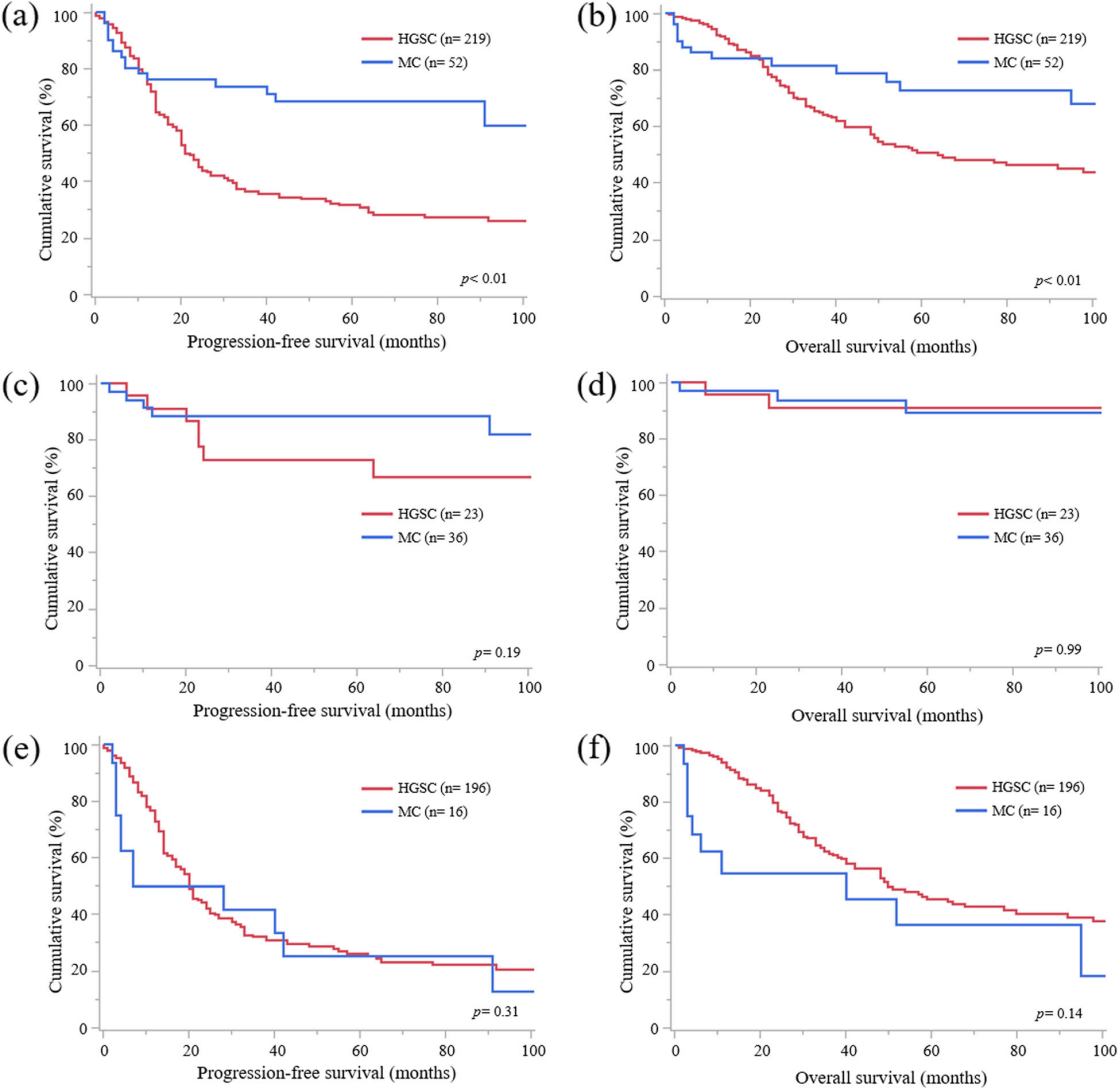
Survival analysis of mucinous carcinoma and high-grade serous carcinoma. **A** Progression-free survival (PFS) curves of all cases of mucinous carcinoma (MC) and high-grade serous carcinoma (HGSC). HGSC has a worse prognosis than MC (*p* < 0.01). **B** overall survival (OS) curves of all cases of MC and HGSC. HGSC has a worse prognosis than MC (*p* < 0.01). **C** PFS curves of cases of MC and HGSC at International Federation of Gynecology and Obstetrics (FIGO) stage I. There is no statistically significant difference in PFS between MC and HGSC (*p* = 0.19). **D** OS curves of cases of MC and HGSC at FIGO stage I. There is no statistically significant difference in OS between MC and HGSC (*p* = 0.99). **E** PFS curves of cases of MC and HGSC at FIGO stages II to IV. There is no statistically significant difference in PFS between MC and HGSC (*p* = 0.31). **F** OS curves of cases of MC and HGSC at FIGO stages II to IV. There is no statistically significant difference in OS between MC and HGSC (*p* = 0.14)

First, survival analysis between MC and HGSC was performed. Compared to cases of HGSC, cases of MC at all stages were diagnosed at younger age (*p* < 0.01) and earlier FIGO stage (*p* < 0.01), less frequently had positive peritoneal cytology (*p* < 0.01), had less residual tumor at primary surgery (*p* < 0.01), and less frequently received taxane-platinum therapy (*p* < 0.01) (Table 1). MC had a better prognosis in terms of PFS (Fig. 2A, *p* < 0.01) and OS (Fig. 2B, *p* < 0.01) than HGSC at all stages. Multivariate analysis revealed that MC at all stages was not a prognostic factor for PFS (hazard ratio [HR] 1.05, *p* = 0.87) or OS (HR 1.54, *p* = 0.20) (Table 2). Moreover, there were no statistically significant differences in PFS (Fig. 2C, *p* = 0.19) and OS (Fig. 2D, *p* = 0.99) between MC and HGSC at FIGO stage I. Additionally, there were no statistical differences in PFS (Fig. 2E, *p* = 0.31) and OS (Fig. 2F, *p* = 0.14) between MC and HGSC at FIGO stages II to IV.

**Table 1:** Characteristics of cases of mucinous carcinoma and high-grade serous carcinoma

Variables	Mucinous carcinoma		Mucinous carcinoma with infiltrative invasion		Mucinous carcinoma with expansile invasion		High-grade serous carcinoma		*p*-value ^a^	*p*-value ^b^	*p*-value ^c^
	*n* =52		*n* = 27		*n* = 25		*n* = 219				
Age (years)											
Median ± SD	49.9 ± 17.7		52.6 ± 17.3		47.0 ± 18.0		58.2 ± 11.5		<0.01	0.11	<0.01
≥60	18	(34.6)	10	(37.0)	8	(32.0)	101	(46.1)	0.16	0.42	0.21
<60	34	(65.4)	17	(63.0)	17	(68.0)	118	(53.9)			
FIGO stage (%)									<0.01	<0.01	<0.01
I	36	(69.2)	16	(59.3)	20	(80.0)	23	(10.5)			
II	3	(5.8)	1	(3.7)	2	(8.0)	18	(8.2)			
III	8	(15.4)	7	(25.9)	1	(4.0)	126	(57.5)			
IV	5	(9.6)	3	(11.1)	2	(8.0)	52	(23.7)			
Peritoneal cytology (%)									<0.01	<0.01	<0.01
Positive	24	(46.2)	14	(51.9)	10	(40.0)	178	(81.3)			
Negative	28	(53.8)	13	(48.1)	15	(60.0)	41	(18.7)			
Residual tumor at PDS (%)									<0.01	<0.01	<0.01
Yes	13	(25.0)	9	(33.3)	4	(16.0)	152	(69.4)			
No	39	(75.0)	18	(66.7)	21	(84.0)	67	(30.6)			
Adjuvant chemotherapy (%)									<0.01	<0.01	<0.01
Taxane-platinum therapy	8	(15.4)	6	(22.2)	2	(8.0)	132	(60.3)			
Platinum-based therapy	26	(50.0)	16	(59.3)	10	(40.0)	65	(29.7)			
Not administered	18	(34.6)	5	(18.5)	13	(52.0)	22	(10.0)			
Response rate (%) ^d^									0.14	0.02	0.99
CR/PR	6	(54.5)	3	(37.5)	3	(100.0)	105	(77.2)			
SD/PD	5	(45.5)	5	(62.5)	0	(0.0)	31	(22.8)			

Abbreviations

SD, standard deviation; FIGO, International Federation of Obstetrics and Gynecology; PDS, primary debulking surgery; CR, complete response; PR, partial response; SD, stable disease; PD, progressive disease

^a^ Mucinous carcinoma vs. high-grade serous carcinoma

^b^ Mucinous carcinoma with infiltrative invasion vs. high-grade serous carcinoma

^c^ Mucinous carcinoma with expansile invasion vs. high-grade serous carcinoma

^d^ One case of mucinous carcinoma with infiltrative invasion, one case of mucinous carcinoma with expansile invasion, and 16 cases of high-grade serous carcinoma with residual tumor at primary debulking surgery did not receive any adjuvant chemotherapy

**Table 2:** Survival analysis for cases of mucinous carcinoma and high-grade serous carcinoma

	Progression-free survival						Overall survival					
	Univariate analysis			Multivariate analysis			Univariate analysis			Multivariate analysis		
Variables	HR (95% CI)		*p*-value	HR (95% CI)		*p*-value	HR (95% CI)		*p*-value	HR (95% CI)		*p*-value
Age (years)												
≥60 vs. <60	1.25	(0.92–1.70)	0.16				1.20	(0.83–1.73)	0.32			
FIGO stage												
I vs. II, III, IV	0.18	(0.09–0.30)	<0.01	0.19	(0.08–0.39)	<0.01	0.10	(0.04–0.22)	<0.01	0.08	(0.03–0.22)	<0.01
Residual tumor at PDS												
Yes vs. No	2.40	(1.70–3.44)	<0.01	1.11	(0.76–1.68)	0.60	2.65	(1.75–4.12)	<0.01	1.05	(0.67–1.70)	0.84
Histological subtype												
MC vs. HGSC	0.40	(0.23–0.65)	<0.01	1.05	(0.57–1.80)	0.87	0.45	(0.24–0.78)	<0.01	1.54	(0.78–2.79)	0.20

Abbreviations

HR, hazard ratio; CI, confidence interval; FIGO, International Federation of Obstetrics and Gynecology; PDS, primary debulking surgery; MC, mucinous carcinoma; HGSC, high-grade serous carcinoma

**Fig. 3:**
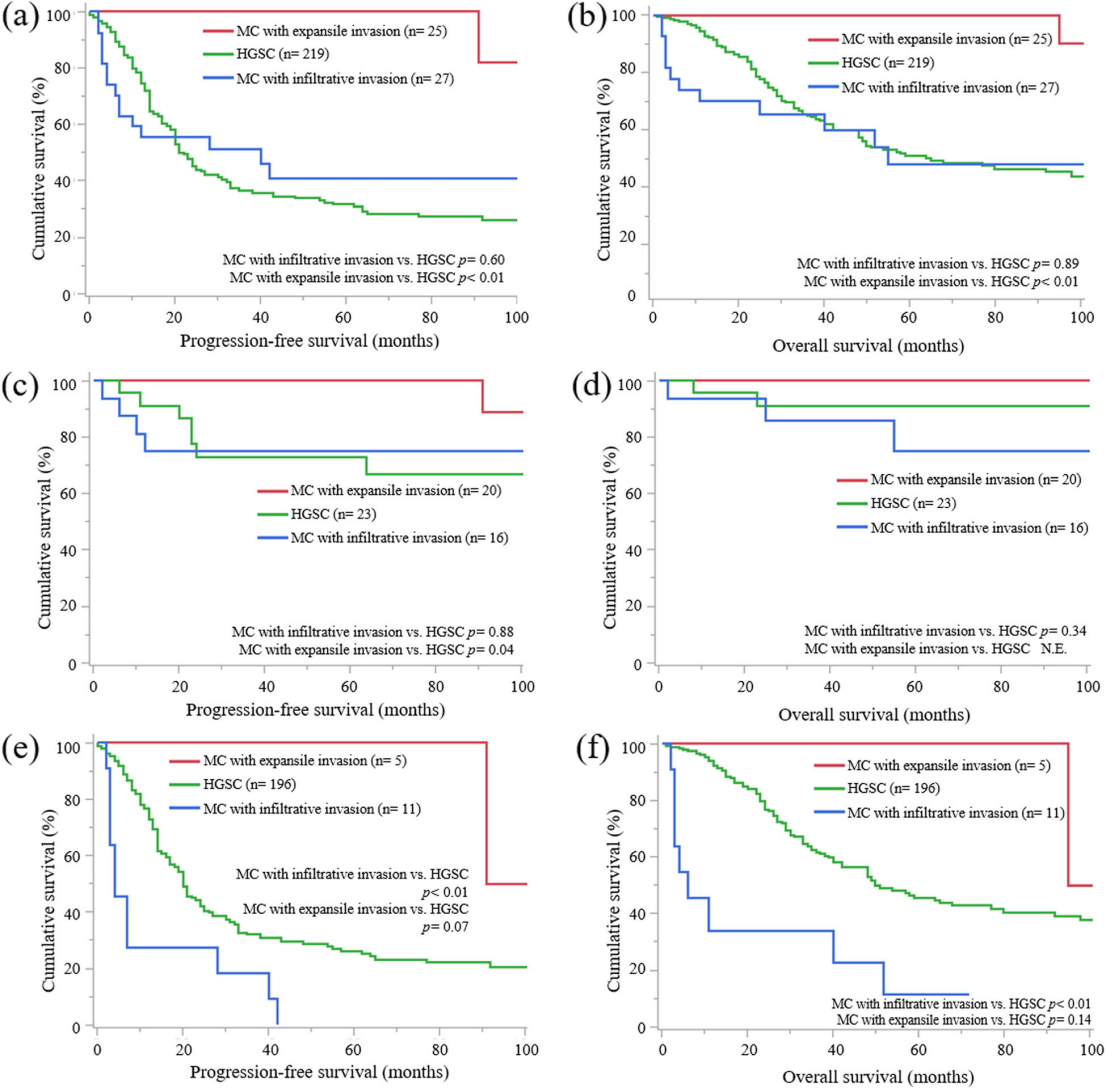
Survival analysis of mucinous carcinoma with infiltrative or expansile invasion and high-grade serous carcinoma. (**A**) Progression-free survival (PFS) curves of all cases of mucinous carcinoma (MC) with infiltrative invasion, MC with expansile invasion, and high-grade serous carcinoma (HGSC). HGSC has a worse prognosis than MC with expansile invasion (*p* < 0.01), and there is no statistically significant difference between MC with infiltrative invasion and HGSC (*p* = 0.60). (**B**) overall survival (OS) curves of all cases of MC with infiltrative invasion, MC with expansile invasion, and HGSC. HGSC has a worse prognosis than MC with expansile invasion (*p* < 0.01), and there is no statistically significant difference between MC with infiltrative invasion and HGSC (*p* = 0.89). (**C**) PFS curves of cases of MC with infiltrative invasion, MC with expansile invasion, and HGSC at International Federation of Gynecology and Obstetrics (FIGO) stage I. MC with expansile invasion has a better prognosis than HGSC (*p* = 0.04), and there is no statistically significant difference between MC with infiltrative invasion and HGSC (*p* = 0.88). (**D**) OS curves of cases of MC with infiltrative invasion, MC with expansile invasion, and HGSC at FIGO stage I. There is no statistically significant difference between MC with infiltrative invasion and HGSC (*p* = 0.34); statistical significance between MC with expansile invasion and HGSC is not evaluable. (**E**) PFS curves of cases of MC with infiltrative invasion, MC with expansile invasion, and HGSC at FIGO stages II to IV. MC with infiltrative invasion has a worse prognosis than HGSC (*p* < 0.01), and there is no statistically significant difference between MC with expansile invasion and HGSC (*p* = 0.07). (**F**) OS curves of cases of MC with infiltrative invasion, MC with expansile invasion, and HGSC at FIGO stages II to IV. MC with infiltrative invasion has a worse prognosis than HGSC (*p* < 0.01), and there is no statistically significant difference between MC with expansile invasion and HGSC (*p* = 0.14)

Second, survival analysis between MC with infiltrative invasion and HGSC was performed. Compared to cases of HGSC, cases of MC with infiltrative invasion at all stages were diagnosed at an earlier FIGO stage (*p* < 0.01), less frequently had positive peritoneal cytology (*p* < 0.01), had less residual tumor at primary surgery (*p* < 0.01), less frequently received taxane-platinum therapy (*p* < 0.01), and had a worse response rate to adjuvant chemotherapy (*p* = 0.02) (Table 1). There were no statistically significant differences in PFS (Fig. 3A, *p* = 0.60) and OS (Fig. 3B, *p* = 0.89) between the two groups at all stages. Furthermore, univariate analysis showed that MC with infiltrative invasion at all stages was not a prognostic factor for PFS (HR 0.89, *p* = 0.60) or OS (HR 1.04, *p* = 0.89) (Table 3). There were no statistically significant differences in PFS (Fig. 3C, *p* = 0.88) and OS (Fig. 3D, *p* = 0.34) between both groups at FIGO stage I, but MC with infiltrative invasion had a worse prognosis in terms of PFS (Fig. 3E, *p* < 0.01) and OS (Fig. 3F, *p* < 0.01) than HGSC at FIGO stages II to IV. Univariate analysis revealed that MC with infiltrative invasion was an independent prognostic factor for PFS (HR 2.83, *p* < 0.01) and OS (HR 3.83, *p* < 0.01) in FIGO stage II to IV.

**Table 3:** Survival analysis for cases of mucinous carcinoma with infiltrative invasion and high-grade serous carcinoma

	Progression-free survival						Overall survival					
	Univariate analysis			Multivariate analysis			Univariate analysis			Multivariate analysis		
Variables	HR (95% CI)		*p*-value	HR (95% CI)		*p*-value	HR (95% CI)		*p*-Value	HR (95% CI)		*p*-value
Age (years)												
≥60 vs. <60	1.11	(0.82–1.52)	0.49				1.07	(0.74–1.54)	0.72			
FIGO stage												
I vs. II, III, IV	0.25	(0.13–0.44)	<0.01	0.28	(0.13–0.54)	<0.01	0.15	(0.05–0.34)	<0.01	0.17	(0.06–0.41)	<0.01
Residual tumor at PDS												
Yes vs. No	1.93	(1.37–2.77)	<0.01	1.15	(0.79–1.74)	0.47	2.09	(1.38–3.26)	<0.01	1.12	(0.72–1.81)	0.64
Histological subtype												
MC with infiltrative invasion vs. HGSC	0.89	(0.49–1.43)	0.60				1.04	(0.54–1.82)	0.89			

Abbreviations

HR, hazard ratio; CI, confidence interval; FIGO, International Federation of Obstetrics and Gynecology; PDS, primary debulking surgery; MC, mucinous carcinoma; HGSC, high-grade serous carcinoma

Third, MC with expansile invasion and HGSC were compared. Compared to cases of HGSC, cases of MC with expansile invasion at all stages were diagnosed at a younger age (*p* < 0.01) and an earlier FIGO stage (*p* < 0.01), less frequently had positive peritoneal cytology (*p* < 0.01), had less residual tumor at primary surgery (*p* < 0.01), and less frequently received taxane-platinum therapy (*p* < 0.01) (Table 1). MC with expansile invasion at all stages had a better prognosis in terms of PFS (Fig. 3A, *p* < 0.01) and OS (Fig. 3B, *p* < 0.01) than HGSC. Multivariate analysis revealed that MC with expansile invasion at all stages was a better prognostic factor for PFS (HR 0.17, *p* < 0.01) and OS (HR 0.18, *p* = 0.03) than HGSC (Table 4). At FIGO stage I, MC with expansile invasion had a better prognosis in terms of PFS than HGSC (Fig. 3C, *p* = 0.04), and OS was not evaluable between the two groups (Fig. 3D). Univariate analysis revealed that MC with expansile invasion at FIGO stage I was a better prognostic factor for PFS than HGSC (HR 0.15, *p* = 0.03). At FIGO stages II to IV, there were no statistically significant differences in PFS (Fig. 3E, *p* = 0.07) and OS (Fig. 3F, *p* = 0.14) between the groups. Univariate analysis showed that MC with expansile invasion at FIGO stages II to IV was a better prognostic factor for PFS than HGSC (HR 0.20, *p* = 0.03).

**Table 4:** Survival analysis for cases of mucinous carcinoma with expansile invasion and high-grade serous carcinoma

	Progression-free survival						Overall survival					
	Univariate analysis			Multivariate analysis			Univariate analysis			Multivariate analysis		
Variables	HR (95% CI)		*p*-value	HR (95% CI)		*p*-value	HR (95% CI)		*p*-Value	HR (95% CI)		*p*-value
Age (years)												
≥60 vs. <60	1.28	(0.92–1.76)	0.14				1.21	(0.82–1.78)	0.34			
FIGO stage												
I vs. II, III, IV	0.16	(0.07–0.30)	<0.01	0.28	(0.12–0.61)	<0.01	0.05	(0.01–0.17)	<0.01	0.10	(0.01–0.34)	<0.01
Residual tumor at PDS												
Yes vs. No	2.26	(1.57–3.33)	<0.01	1.17	(0.78–1.79)	0.46	2.61	(1.67–4.24)	<0.01	1.15	(0.73–1.91)	0.56
Histological subtype												
MC with expansile invasion vs. HGSC	0.08	(0.01–0.24)	<0.01	0.17	(0.03–0.58)	<0.01	0.06	(0.003–0.25)	<0.01	0.18	(0.01–0.86)	0.03

Abbreviations

HR, hazard ratio; CI, confidence interval; FIGO, International Federation of Obstetrics and Gynecology; PDS, primary debulking surgery; MC, mucinous carcinoma; HGSC, high-grade serous carcinoma

## Discussion

In our study, univariate analysis revealed that MC was a better prognostic factor for PFS and OS than HGSC at all FIGO stages, but multivariate analysis did not demonstrate this. In addition, there were no statistical differences in PFS and OS between patients with MC with infiltrative invasion and those with HGSC at all stages or at stage I. However, univariate analysis showed that MC with infiltrative invasion at FIGO stages II to IV had worse PFS and OS than HGSC. In addition, multivariate analysis showed that MC with expansile invasion was a better prognostic factor for PFS and OS than HGSC at all stages.

Many studies have compared MC and HGSC, but these studies included cases of MC with infiltrative invasion as well as those of MC with expansile invasion defined firstly by 2014 WHO criteria [17–28, 32]. To our knowledge, this is the first report comparing MC with infiltrative, MC with expansile invasion, and HGSC.

According to the previous studies, MC is diagnosed at a younger age and an earlier FIGO stage and has worse chemosensitivity than HGSC [3, 17–20, 33]. In our study, MC with expansile invasion was diagnosed at a younger age and an earlier FIGO stage than HGSC. Hence, a lower response to chemotherapy was associated with MC with infiltrative patterns. However, there are disagreements regarding the prognosis of MC compared with that of HGSC [17–27]. In our study, MC with infiltrative invasion had a worse prognosis than HGSC at advanced FIGO stages, and MC with expansile invasion had a better prognosis than HGSC at all stages. Because previous study demonstrated that MC with expansile invasion had similar prognosis with ovarian mucinous borderline tumor, the biological behavior of MC with expansile invasion is different from that of MC with infiltrative invasion [34]. Therefore, the inconsistent findings reported by previous studies may be due to different proportions of cases of MC with expansile invasion and infiltrative invasion included. Also, we thought the low response rate to chemotherapy of MC with invasive pattern might be strongly associated with biological behavior. Future studies on MC should examine MC with expansile invasion and MC with infiltrative invasion separately.

HGSC has been frequently associated with breast cancer gene (BRCA) 1 or BRCA2 mutations, and poly (adenosine diphosphate-ribose) polymerase inhibitors have been shown to be effective for cases with HGSC harboring BRCA mutations [35–38]. Hence, BRCA1/2 mutations were not observed in MC. In addition, compared with HGSC, allelic loss at distal 8p were less frequently observed in MC and expression of transcription factor GATA-4 were more often observed in MC. Thus, the genetic information might be helpful to distinguish MC from HGSC [28, 36]. However, although treatments for HGSC were developed based on genetic background, no new treatments have been developed for MC. Recent studies have shown that more cases with MC with infiltrative invasion was positive for cytokeratin 5/6, cluster of differentiation 24, and epidermal growth factor receptor than those with MC with expansile invasion, while more cases with MC with expansile invasion was positive for human epidermal growth factor receptor type 2 compared with those with MC with infiltrative invasion [12, 39]. Furthermore, recent study has also shown that positive expression of cytokeratin 5/6 was associated with worse PFS and positive expression of epidermal growth factor receptor was associated with worse PFS and OS for MC [12]. The information will be useful as the candidate of the new therapy. Furthermore, the biomarker might be useful to not only extinguish MC with infiltrative invasion form MC with expansile invasion but also predict the prognosis.

This study has some limitations. This retrospective study included a small sample size from a single institution. Although our study included a small number of cases, our study had a larger sample size than that several previous studies. Moreover, this study did not adapt the sectioning and extensively examining the fimbria (SEE-FIM) protocol for all cases with HGSC because cases before the SEE-FIM protocol was designed were included in our study. Therefore, histological examination of the fallopian tube might be insufficient. Further large-scale studies are needed to confirm the clinical significance of MC in the future. Furthermore, although the treatment options for MC with infiltrative invasion have already established in the 2019 European Society for Medical Oncology and European Society of Gynaecological Oncology consensus conference [40], we believed the information of our study will be useful for future studies of the treatment for MC because the development of the new treatment to improve the prognosis will be needed.

## Conclusions

The clinicopathological review of MC with infiltrative invasion, MC with expansile invasion, and HGSC revealed that MC with infiltrative invasion had worse prognostic features than HGSC at advanced stages, and further studies on the treatment of MC are needed.

## Data Availability

The datasets used and/or analysed during the current study are available from the corresponding author on reasonable request.

## Abbreviations

MC: mucinous carcinoma
HGSC: high-grade serous carcinoma
FIGO: International Federation of Gynecology and Obstetrics
PFS: progression-free survival
OS: overall survival
HR: hazard ratio
EOC: epithelial ovarian carcinoma
BRCA: breast cancer gene
SEE-FIM: the sectioning and extensively examining the fimbria
